# Inactivation of *Listeria* and *E. coli* by Deep-UV LED: effect of substrate conditions on inactivation kinetics

**DOI:** 10.1038/s41598-020-60459-8

**Published:** 2020-02-25

**Authors:** Yifan Cheng, Hanyu Chen, Luis Alberto Sánchez Basurto, Vladimir V. Protasenko, Shyam Bharadwaj, Moududul Islam, Carmen I. Moraru

**Affiliations:** 1 Department of Food Science, Cornell University, Ithaca, NY 14850 USA; 2 Universidad Autónoma de Querétaro, Santiago de Querétaro, Mexico; 3 Electrical and Computer Engineering, Cornell University, Ithaca, NY 14850 USA

**Keywords:** Applied microbiology, Bacteria, Food microbiology, Lasers, LEDs and light sources

## Abstract

Irradiation with deep-ultraviolet light-emitting diodes (DUV LEDs) is emerging as a low energy, chemical-free approach to mitigate microbial contamination, but the effect of surface conditions on treatment effectiveness is not well understood. Here, inactivation of *L. innocua* and *E. coli* ATCC25922, as examples of Gram-positive and Gram-negative bacteria, respectively, by DUV LED of 280 nm wavelength was studied. Surface scenarios commonly encountered in environmental, clinical or food processing environments were used: nutrient rich surfaces, thin liquid films (TLF), and stainless steel surfaces (SS). DUV LED exposure achieved 5-log reduction for both strains within 10 min in most scenarios, except for TLF thicker than 0.6 mm. Inactivation kinetics in TLF and on dry SS followed the Weibull model (0.96 ≤ *R*^2^ ≤ 0.99), but the model overestimated inactivation by small-dose DUV on wet SS. Confocal microscopy revealed *in situ* that bacteria formed a dense outer layer at the liquid-air interface of the liquid droplet, protecting the cells inside the droplet from the bactericidal DUV. This resulted in lower than anticipated inactivation on wet SS at small DUV doses, and deviation from the Weibull model. These findings can be used to design effective DUV LED disinfection strategies for various surface conditions and applications.

## Introduction

Persistence of pathogens on material surfaces often causes severe consequences, including infections in dental offices and hospitals^1^, or transfer of pathogenic or spoilage microorganisms from food contact surfaces to food products in food processing facilities and food service environments^2^. Exposing surfaces contaminated by microorganisms to ultraviolet (UV) of wavelength 100–280 nm has been established as an effective disinfection method, often used as an alternative to or in tandem with chemical disinfection methods. Mercury lamps are currently the most commonly used source of UV light. Yet, according to the Minamata Convention on Mercury^3^, signed in 2013, manufacturing and trading of mercury-containing lamps for general lighting purposes will be disallowed after 2020, to reduce and eliminate the adverse effects of mercury on human health and the environment. This agreement accelerated the efforts for the development of alternatives to mercury lamps.

Light-emitting diodes that emit light in the UV range (UV LEDs) present several advantages compared to mercury lamps, including the lack of toxic mercury, device compactness and flexible designs, zero warm-up time^4,5^, high durability, monochromatic light emission at specific wavelength^6^, wavelength diversity, possibility of pulsed illumination, and the capability of maintaining relatively high activity at cold temperatures (e.g. refrigeration)^5^. UV LEDs are also known for their low heat emission in the form of IR radiation^7^, which enables applications that demand high UV fluence while preventing heating over long periods of time. Recent progress in improving the light-extraction efficiency of UV LEDs in the range 200–300 nm has increased the external quantum efficiency beyond 20%, approaching the 30–40% efficiency range of low-pressure UV lamps^8^.

This also resulted in increasing interest in substituting mercury lamps with UV LEDs for bacteria inactivation. Successful applications of deep UV LEDs (DUV-LEDs), which emit light in the wavelength range 200–300 nm, have recently emerged in the healthcare industry (e.g. disinfection of endoscopes, breathing circuits, and respirators), agriculture (e.g. disinfection of irrigation and feed water), packaging plants (e.g. air disinfection), food service (e.g. food contact surfaces disinfection), and homes (e.g. disinfection of cell phone surfaces and drinking water).

One limitation of using DUV LED as a bactericidal technology is the short penetration depth of UV light, which impairs effectiveness in inactivating bacteria that reside deeper than the surface of solid or liquid media. To mitigate this drawback, previous work on liquid disinfection via DUV adopted stirring^5,9–11^ or turbulent flow^12,13^, to facilitate access of DUV to the target microorganisms. Notwithstanding its limited penetration depth, DUV treatment is well-suited for disinfecting surfaces, either in dry conditions or in the presence of liquid droplets or thin liquid films. Both scenarios are ubiquitous in environmental applications, the food industry and healthcare industry, yet knowledge on the effectiveness of DUV LED inactivation kinetics of bacteria under such conditions is limited.

Another important aspect that must be considered is the potential reactivation of UV-injured bacterial cells post DUV LED treatment^11^, which could have tremendous safety implications, as it can diminish the overall effectiveness of the treatment.

To address the current knowledge gaps in the area of bactericidal effectiveness of DUV LEDs, in the present study the germicidal effectiveness and post-UV repair of bacterial cells treated with 280 nm wavelength DUV LEDs was evaluated, for various substrate scenarios. A custom-made DUV LED treatment panel with a peak emission wavelength at 280 nm was used, both because 280 nm has higher electrical efficiency than the UV wavelength typically used for disinfection (e.g. 254 nm), and because it was shown to have higher germicidal efficiency at the same energy expenditure, without excessive heat generation during the operation^14^. *Escherichia coli* ATCC 25922 and *Listeria innocua* were selected as challenge microorganisms because they are proven surrogates for UV-based treatments for pathogenic *E. coli* O157:H7^12,15^ and *L. monocytogenes*^16^, respectively.

The findings of this study provide insights into the kinetics and factors of influence for microbial inactivation by DUV, which could be used to design effective and efficient DUV LED surface disinfection applications.

## Results

### Inactivation of bacteria streaked on nutritive agar

**Figure 1:**
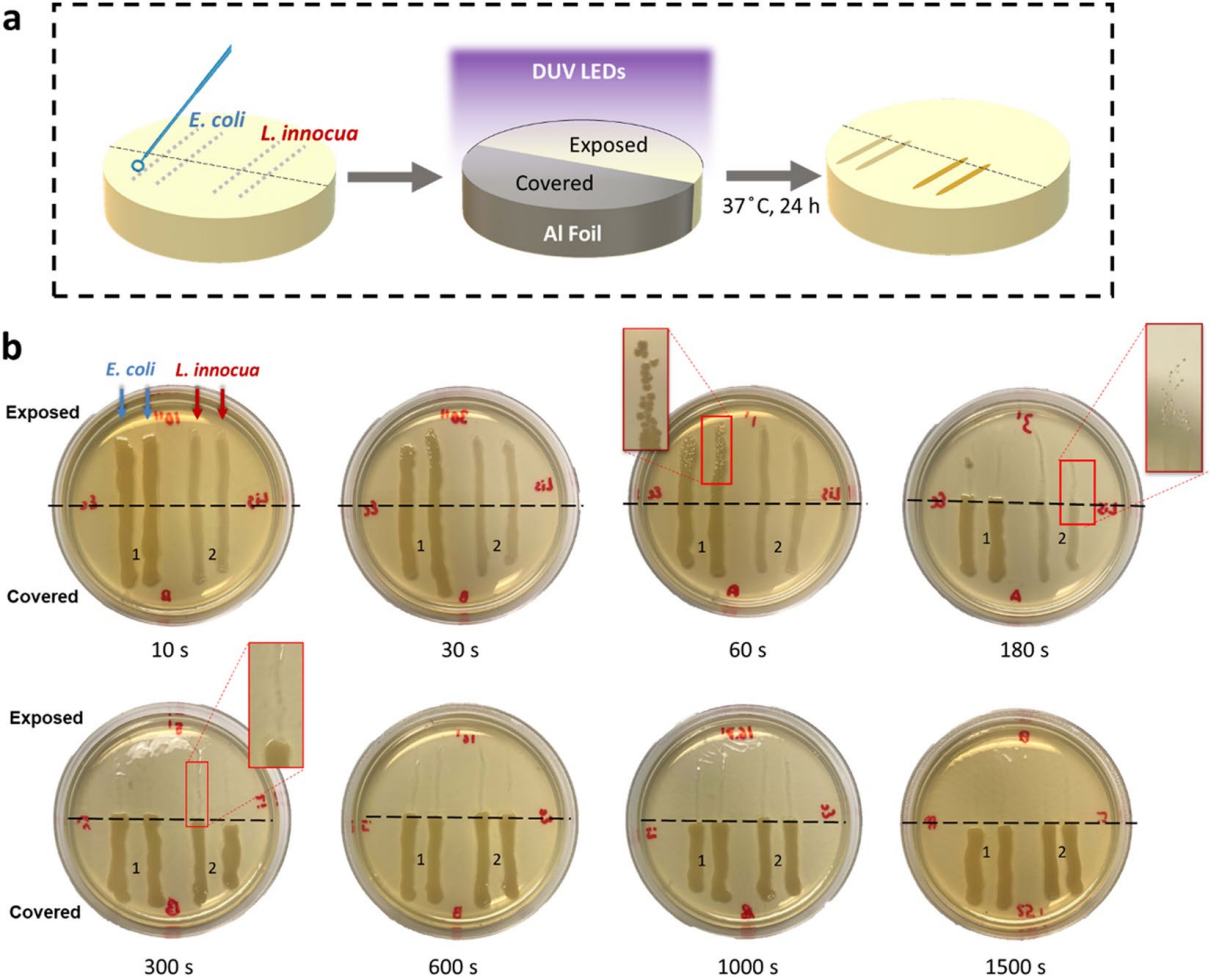
Qualitative evaluation of DUV LED inactivation of *E. coli* and *L. innocua* on TSA agar plates, which mimic nutrient-rich surfaces. (**a**) Schematic flow chart of the experimental steps. (**b**) Photos of the agar plates after 24 h incubation, showing the differential growth patterns of bacteria streaks with and without exposure to DUV LEDs. The agar area below the black dashed line was protected from exposure to DUV.

The qualitative results showed a significant reduction in the culturable population of *E*. *coli* and *L*. *innocua* cells on nutrient-rich surfaces exposed to DUV LED treatments. A gradual decrease in viable bacterial cells was achieved within 1 min of DUV LED exposure for both strains, as revealed by the gradual decrease in the density of colonies comprising the streaks after incubation (Fig. 1b). After 3 min of continuous DUV LED exposure, no visible *E*. *coli* colonies were observed on the area not covered by aluminum foil. Scattered *L*. *innocua* colonies were observed near the border between exposed and covered areas, possibly due to partial blockage of DUV by the aluminum foil. After 5 min, no visible colonies were found along the streaking traces on the exposed area, for either strain. On the areas covered with aluminum foil, both strains grew into dense, continuous stripes along the streaking traces. Their growth was unaffected even for the longest treatment time tested. It should also be noted that the 24-h incubation on nutrient-rich agar surfaces at optimal growth temperature (37 °C) provided an ideal condition for post-UV reactivation of any damaged cells to occur^17^, yet no reactivation was observed beyond 5 min of DUV exposure (11.88 mJ/cm^2^).

### Inactivation of bacteria in thin liquid films of various thickness

**Figure 2:**
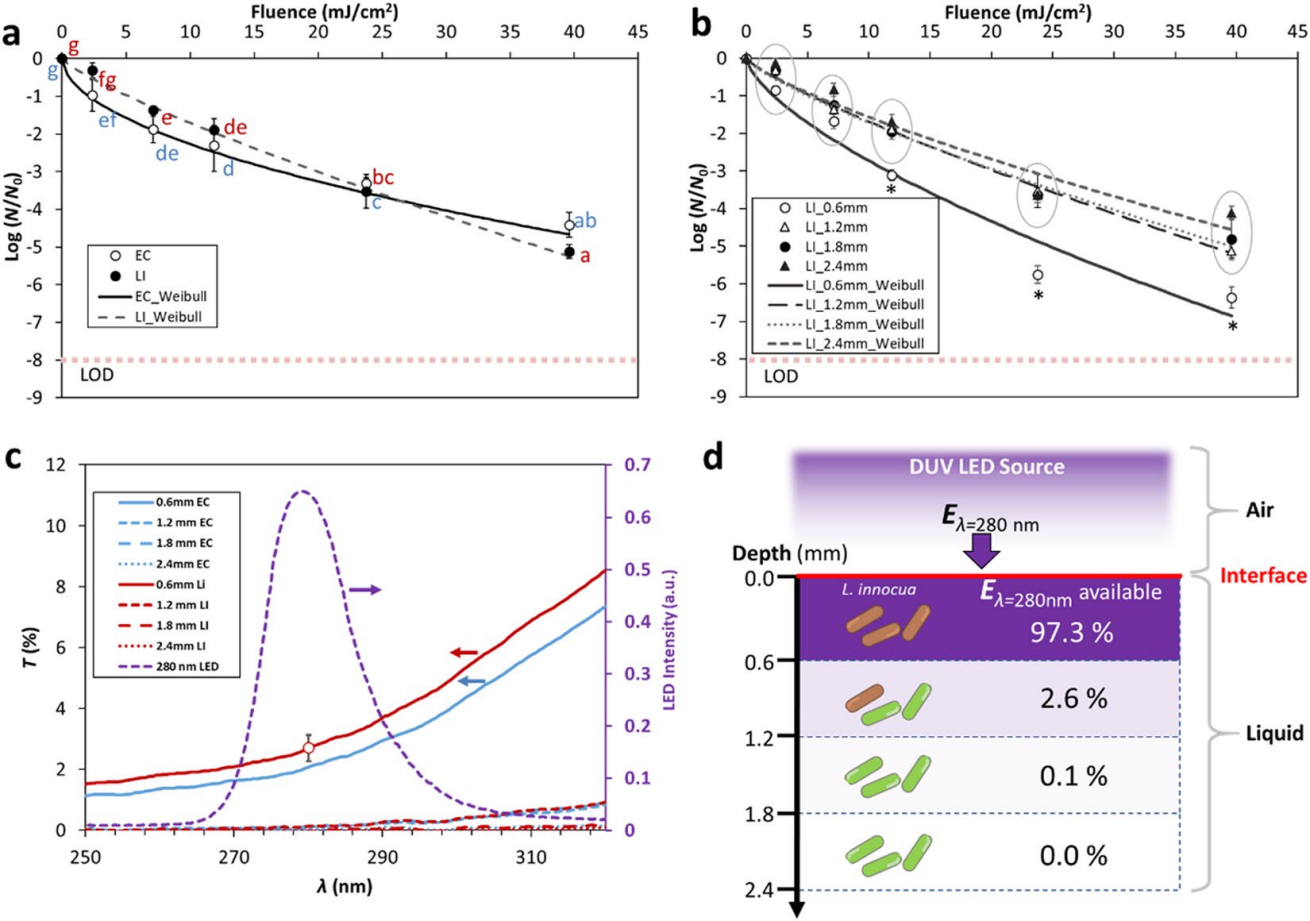
DUV LED inactivation kinetics of *E. coli* (EC) and *L. innocua* (LI) suspended in thin liquid films. (**a**) Comparison between the inactivation kinetics of *E. coli* and *L. innocua* suspended in 1.2 mm thick BPB buffer. Different letters denote significant differences (*p* < 0.05); inactivation data for EC (blue) and LI (red) were analyzed together. Limit of detection (LOD) is denoted by a dotted line. (**b**) Inactivation kinetics of *L. innocua* suspended in BPB buffer of various thickness. Asterisks denote data points that are significantly different from the others at the same DUV exposure doses (*p* < 0.05), whereas circles denote clusters of data points that are not significantly different (*p* > 0.05). (**c**) Transmittance (T%, left axis) of *E. coli* and *L. innocua* suspensions of various thickness over the light spectrum emitted by the DUV LEDs (intensity, right axis). The horizontal arrows point to the relevant axes for each of the curves. (**d**) Percentage of the incident 280 nm energy (*E*_*λ*=280_) available for inactivation at different depth segments of a *L. innocua* suspension. Error bars in (**a**), (**b**), and (**c**) represent stand**a**rd deviations.

For all TLF thicknesses tested, survivor counts decreased nonlinearly with increasing DUV dose emitted by the LEDs. The inactivation kinetics of *E*. *coli* and *L*. *innocua* with TLF thickness of 1.2 mm were determined, which allowed a direct comparison of the responses of the two strains to DUV. Figure 2a shows that overall there were no significant differences in inactivation between the two strains (*p* > 0.05, Fig. 2a).

To explore the effect of TLF thickness on inactivation, *L*. *innocua* suspensions with thickness ranging from 0.6 mm to 2.4 mm were exposed to DUV. The inactivation curves indicate that DUV LED had a much higher efficiency against *L*. *innocua* in the 0.6 mm liquid film than in the thicker TLFs, particularly at DUV fluence>11.88 mJ/cm^2^. For the TLFs with thickness above 1.2 mm, the inactivation curves largely overlapped (Fig. 2b) and no significant differences in log reduction values were found (*p* > 0.05). This result was corroborated by the Weibull model parameters (Table 1), since the scale and shape of the inactivation curves for cells suspended in 0.6 mm thin film were significantly different from the rest (*p* < 0.05). To rule out the possible effect of water evaporation from the TLF on the results, the percent weight loss due to evaporation was determined. For the 0.6 mm, 1.2 mm, 1.8 mm, and 2.4 mm films, the weight losses over 1200 s of DUV-LED exposure were 9.1%, 4.2%, 3.0%, and 2.0%, respectively (Supplementary Fig. S2). This indicates that water evaporation was minor during the DUV LED treatment, and drying can be excluded as a possible reason for cell death.

**Table 1:** Weibull model parameters for the DUV LED inactivation kinetics of *L. innocua* suspended in liquid film of various thickness.

Liquid Film Thickness (mm)	Scale Parameter *α*	Shape Parameter *β*	*R*^2^
0.6	−0.58 ± 0.05 **a**	0.67 ± 0.03 **A**	0.96
1.2	−0.26 ± 0.02 **b**	0.82 ± 0.04 **B**	0.99
1.8	−0.28 ± 0.04 **b**	0.78 ± 0.05 **B**	0.98
2.4	−0.26 ± 0.03 **b**	0.78 ± 0.03 **B**	0.98

To better understand the effect of bacteria suspension thickness on DUV transmission, transmittance spectra through *E. coli* and *L. innocua* suspensions were determined. Generally, the light transmission through the bacterial suspensions was smaller at shorter wavelengths. Figure 2c shows the light transmission of in the spectral range of the DUV-LED source, and Supplementary Fig. S1 shows transmission over an expanded spectral range (220 nm to 900 nm). In Fig. 2c, the emission spectrum of the LEDs used was superimposed onto the transmittance spectra for direct comparison. Within the half width of the peak (i.e. 273 nm–288 nm), transmittance (*T*) was lower than 4% at any given wavelength, even for the thinnest bacteria suspension (0.6 mm). *T* decreased significantly with the increase in thickness, with *T* < 0.4% for the 1.2 mm suspension and *T* < 0.1% through the 1.8 mm and 2.4 mm suspensions.

The transmittance spectra provide insight into the availability of the bactericidal UV through the bacterial cell suspensions. To schematically represent this, a liquid film was virtually compartmentalized into 0.6 mm thick layers (Fig. 2d). In this representation, it was assumed that bacterial cells are uniformly distributed throughout the thickness of the liquid film. This is a simplification, as aggregates of cells can occur at the air-liquid interface, as it will be discussed later. For *L. innocua* suspensions, it was estimated that 2.7% of the incident 280 nm UV energy (*E*_λ=280nm_) is still available after passing through the first 0.6 mm of suspension, suggesting that 97.3% of the energy is lost due to the absorption by the *L. innocua* cells and the BPB medium. The transmittance of *E*_λ=280nm_ through BPB alone was estimated to account for absorption of only 0.4% of *E*_λ=280nm_ (Supplementary Fig. S1), meaning that the *L. innocua* cells in the suspension were responsible for more than 99% of the *E*_λ=280nm_ absorbed. A schematic of the decrease in the available bactericidal energy *E*_λ=280nm_ with increasing suspension depth is represented in Fig. 2d.

### Inactivation of bacteria on stainless steel surfaces under wet and dry conditions

**Figure 3:**
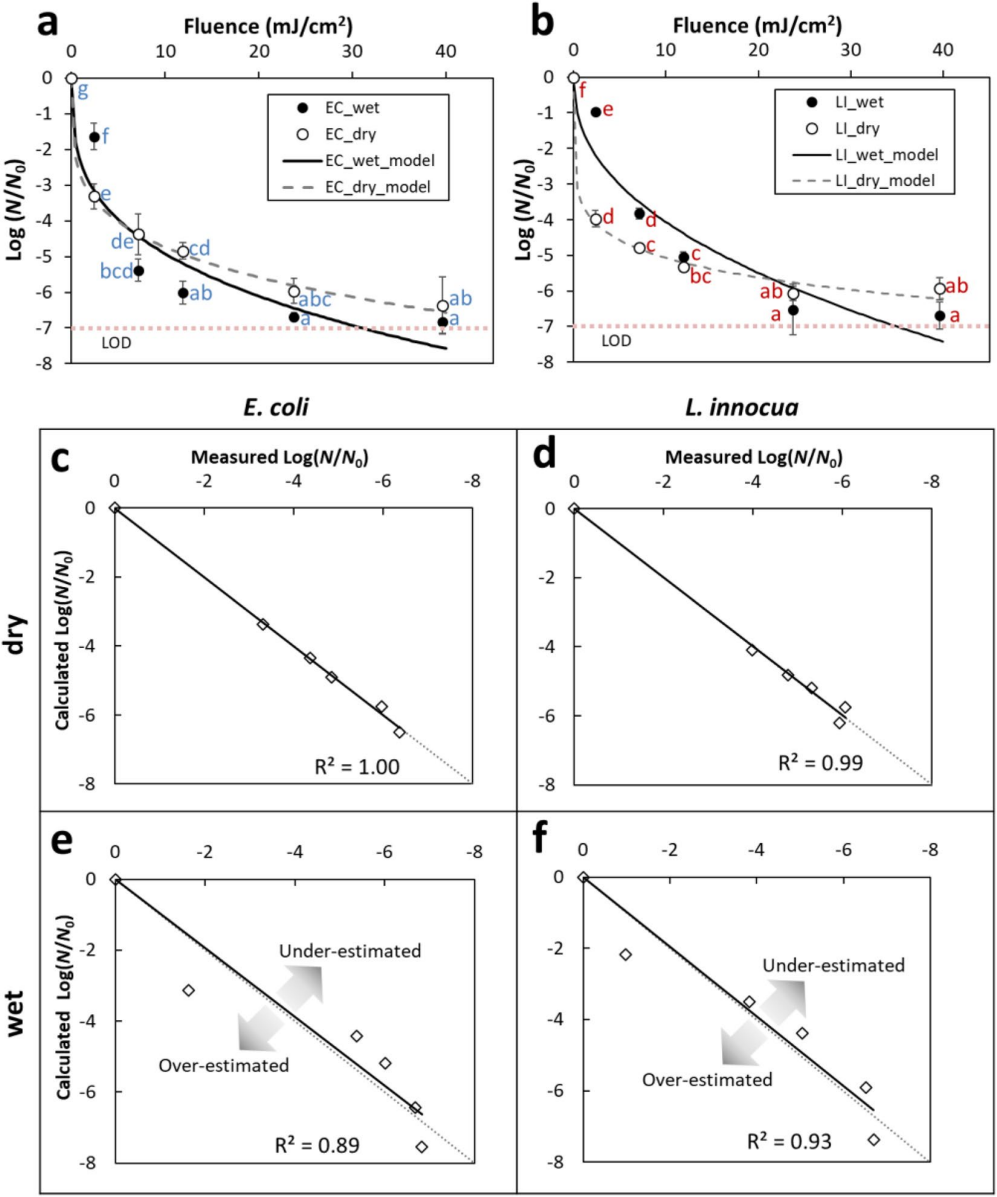
DUV LED inactivation kinetics of *E. coli* (**a**) and *L. innocua* (**b**) on SS coupons in the wet and the dry condition. Error bars represent standard deviations. The limit of detection (LOD) of the assay is denoted by dotted line. Different letters denote significant differences (ANOVA, *p* < 0.05) (data for the two strains was analyzed separately). Fitted vs. measured plots for *E. coli* and *L. innocua* in the dry (**c**,**d**) and the wet condition (**e**,**f**), highlighting the goodness-of-fit for each situation. Arrows in (**e**) and (**f**) suggest over- and under-estimation by the Weibull model.

The presence of liquid droplets considerably reduced the level of inactivation for both *E. coli* (Fig. 3a) and *L. innocua* compared to the inactivation on air-dried SS surfaces (Fig. 3b), especially within the first 60 s of exposure (2.38 mJ/cm^2^ fluence). This effect was more pronounced for *L. innocua*. However, the inactivation curves on dry SS (dashed lines) tend to reach a plateau earlier than on wet SS (solid lines), for both strains (Fig. 3a,b). This was also reflected in the smaller values of the Weibull shape parameters, which are indicative of concave inactivation curves, for dry compared to wet SS (Table 2). A crossover of the wet and dry inactivation curves occurred at a cumulative dose of 7.13 mJ/cm^2^ (2 min treatment) for *E. coli* and at 22.18 mJ/cm^2^ (7 min treatment) for *L. innocua*, respectively. Inactivation curves for both the wet and dry SS reached a plateau around 6–7 log reduction, after a cumulative fluence >20 mJ/cm^2^ (~5 min exposure). The plateau inactivation values were not significantly different between the wet and the dry conditions for either *E. coli* or *L. innocua* (*p* > 0.05).

**Table 2:** Weibull model kinetic parameters for the DUV LED inactivation of *L. innocua* and *E. coli* on stainless steel metal coupons under wet and dry conditions.

Bacteria	Substrate Conditions	Scale Parameter *α*	Shape Parameter *β*	*R*^2^
*E. coli*	Wet	−2.40 ± 0.18 **b**	0.31 ± 0.03 **B**	0.89
*E. coli*	Dry	−2.76 ± 0.47 **b**	0.23 ± 0.06 **AB**	1.00
*L. innocua*	Wet	−1.50 ± 0.10 **c**	0.43 ± 0.03 **C**	0.92
*L. innocua*	Dry	−3.61 ± 0.24 **a**	0.15 ± 0.03 **A**	0.99

The inactivation data was fitted using the Weibull model, and an excellent agreement between measured and predicted Log(*N*/*N*_0_) values was obtained for the dry coupons for both strains, with *R*^2^ = 1.00 and 0.99 for *E. coli* and *L. innocua*, respectively (Fig. 3a–d, Table 2). In case of the wet surfaces the Weibull model did overestimate both the initial inactivation values (dose <2.38 mJ/cm^2^) and the plateau values (dose ≥39.60 mJ/cm^2^), which resulted in weaker strong model fit compared to the dry conditions (R^2^ = 0.90 and 0.93 for *E. coli* and *L. innocua*, respectively). Some of the possible reasons for the weaker predictability of the inactivation data under wet conditions will be discussed next.

### Distribution of bacterial cells at the liquid-air and solid-air interfaces

**Figure 4:**
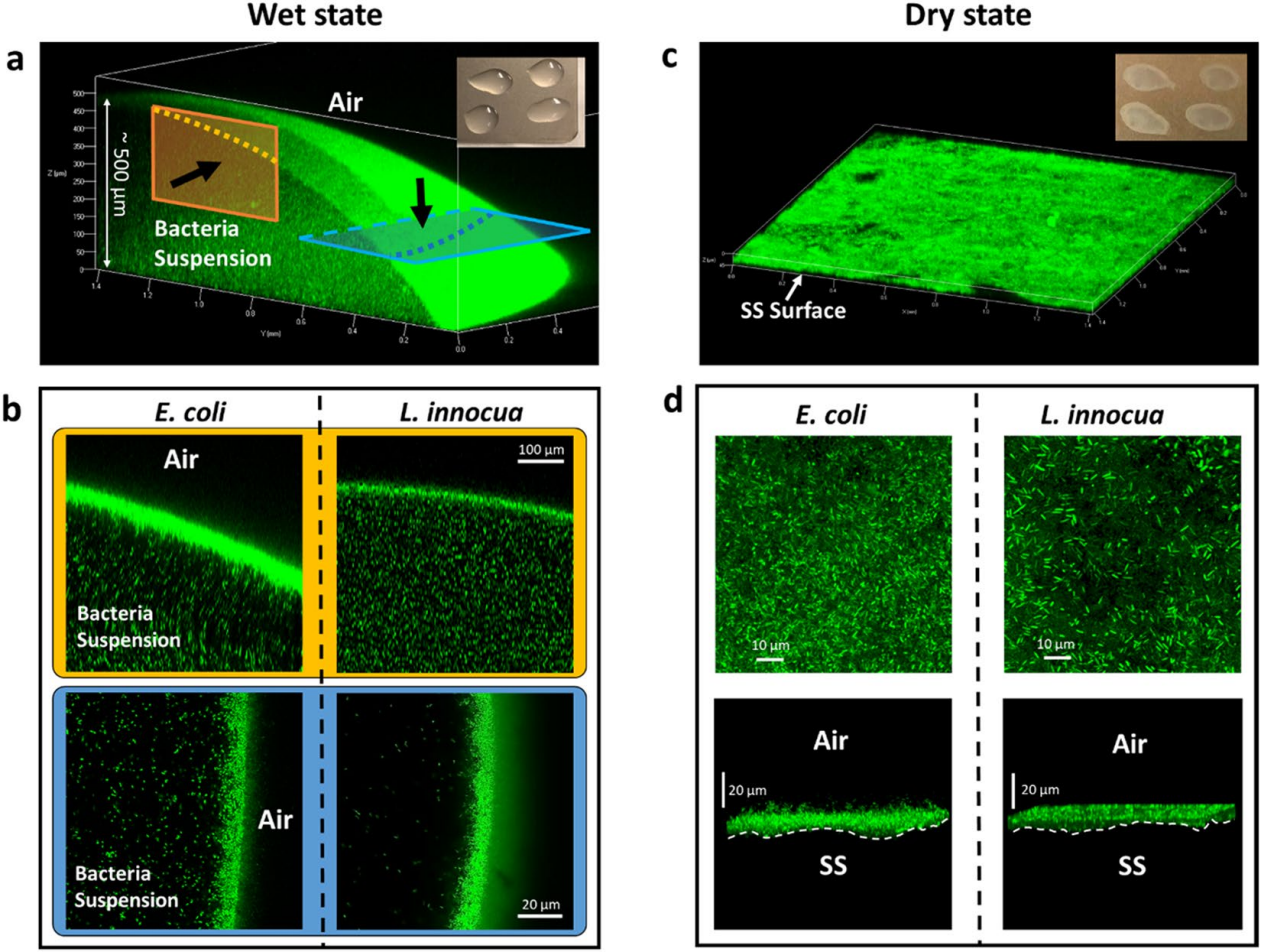
Distribution of untreated *L. innocua* and *E. coli* cells on SS coupons in the wet and the dry state visualized using confocal microscopy. (**a**,**c**): a typical macroscopic view of the fluorescent bacterial cells in the wet (**a**) and the dry (**c**) state. The insets are photos of the coupons in the respective states. (**b**) Cross-sectional views of the top (corresponding to the yellow panel in (**a**)) and the side (blue panel) of typical bacteria-containing droplets on SS coupons in the wet state. Black arrows denote the direction of observation. (**d**) Top-down views (top panel) and side views (bottom panel) of typical pellets of bacteria on SS coupons in the dry state.

Confocal microscopy imaging of a quarter of a bacteria-containing droplet revealed that the distribution of bacterial in the radial direction is not uniform. As seen in Fig. 4a, within each horizontal plane of the 3D reconstruction of the droplet substantially higher fluorescence intensity, which corresponds to higher cell density, was found at the liquid-air interface of the droplet compared to the interior of the droplet. The gradual weakening of fluorescence intensity from the side of the droplet to its apex was likely an artifact caused by a combination of reduced laser power further away from the source and possibly photobleaching. To better visualize the details of the distribution of cells near the liquid-air interface, representative zoomed-in single-slice images in the x-y and x-z planes are shown in Fig. 4b. Strikingly, densely packed, multilayered bacteria cells of *E. coli* (left panel) and *L. innocua* (right panel) made up a dome-like outer shell at the liquid-air interface of the droplet, while the inner core of the liquid droplet contained planktonic cells. In Fig. 4b, the yellow panel shows images taken at the apex of the droplet, while the blue panel shows images taken at the side of the droplet. The horizontal slices in Fig. 4b revealed a smooth outward facing bacteria layer and a less defined, more diffuse appearance of this layer towards the liquid. This is particularly clear in the blue panel. This suggests that bacterial cells tend to preferentially align at the liquid-air interface. After 3 min of equilibration and less than 15 min of total imaging time, the average thickness of the resulting interfacial bacterial cell shells amounts to about 15 µm. Considering that the average length of an *E. coli* cell is 2.22 µm and diameter 0.64 µm, and those of *L. innocua* cells are 1.26 µm and 0.52 µm, respectively (Supplementary Table S1), such a shell is estimated to consist of 10 to 20 layers of bacterial cells in its thickness. As shown in Fig. 2a,b, highly concentrated bacteria suspensions are extremely effective in dampening the incident DUV irradiance. Therefore, it can be inferred that the high cell density of the outer shells of bacteria containing droplets is blocking DUV and is effectively preventing the radiation from reaching the inner planktonic cells.

The thickness (up to 500 µm) of the liquid droplets forms a sharp contrast with the flat morphology of the dry bacteria pellets on SS surfaces, which are typically less than 20 µm thick (Fig. 4c). In the dry state scenario, both *E. coli* and *L. innocua* cell lawns exhibit rather homogenous distribution within the horizontal plane (Fig. 4d, top row) and dense packing in z direction (Fig. 4d, bottom row). The thickness of the bacteria pellet was estimated to contain 10–20 cells in the z direction, which is similar to the outer shell of droplets at the liquid-air interface in the wet scenario.

### Bacteria distribution at the liquid-air interface: a thermodynamic model

**Figure 5:**
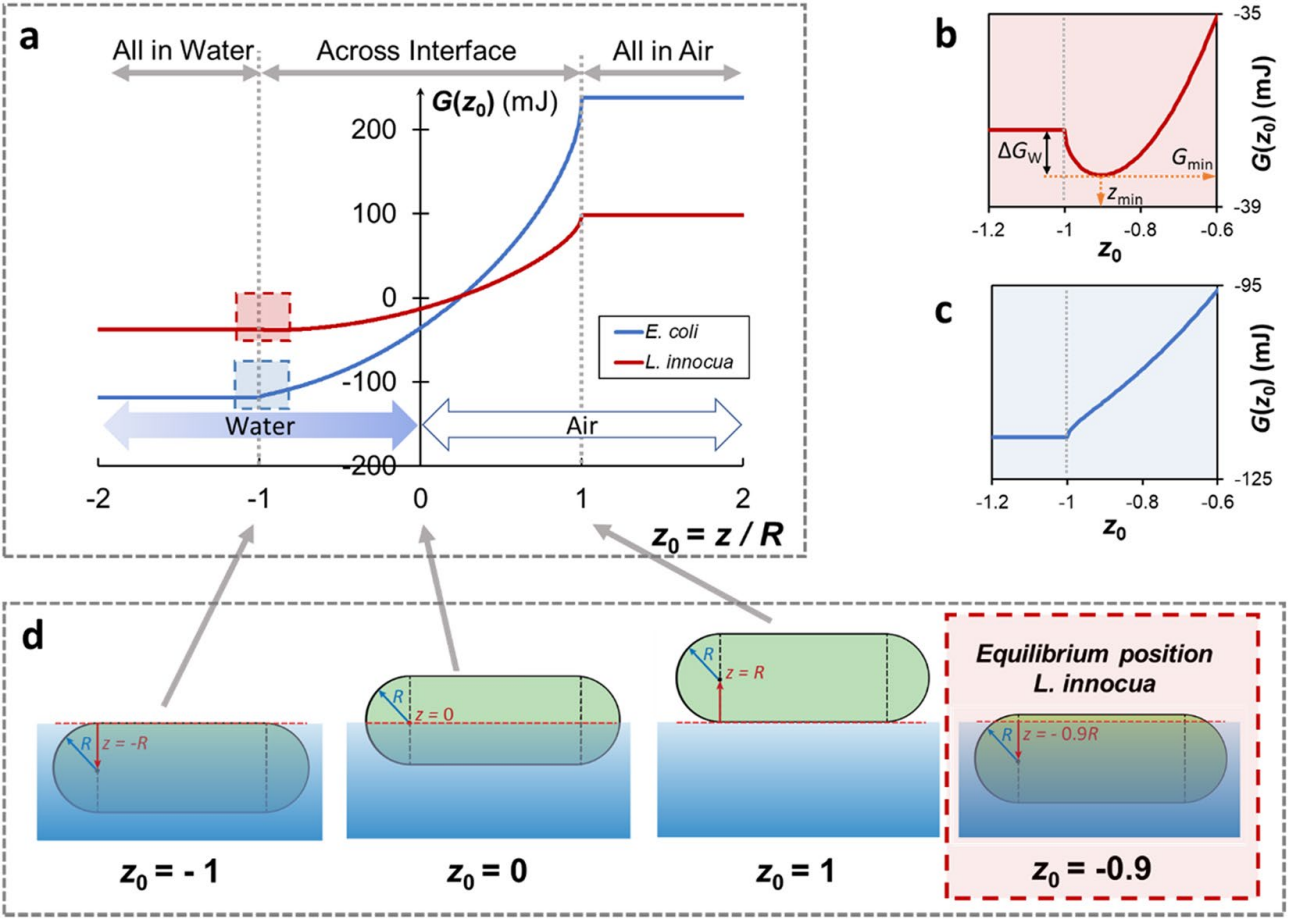
A thermodynamic model of bacterial distribution at the water-air interface. (**a**) System surface energy, *G*, as a function of the relative position, *z*_0_, of an *E. coli* (EC, blue) or *L. innocua* cell (LI, red). *z*_0_ is defined as the vertical coordinate of the center of the cell with regard to the water level, *z*, normalized by the radius of the cell, *R*. (**b**) and (**c**): enlarged view of the energy curves at the ‘escape-to-water’ point (*z*_0_ = −1) for *L. innocua* (red box) and *E. coli* (blue box), respectively. ∆*G*_w_ is the energy barrier a cell needs to overcome to escape into the bulk water phase from the equilibrium position, *z*_min_. (**d**) Schematic illustration of various positions of a cell relative to the interface. The equilibrium position for *L. innocua* (*z*_0_ = −0.9) is highlighted by the red dashed square.

To understand the driving forces behind the aggregation of both bacteria strains at the liquid-air interfaces, a thermodynamic model was developed. The total surface energy (*G*) of the bacterium-water-air system was calculated as a function of a dimensionless coordinate ${z}_{0}$, defined as the vertical coordinate of the center of the cell with regard to the water level, z, normalized by its radius, R (Fig. 5). As the position of a bacterium cell changes from fully immersed in water *z*_0_ ≤ −1, to partially immersed ($-1 < {z}_{0} < 1$), to fully in air (${z}_{0}\ge 1$), the total surface energy of the system appears to increase for both strains, but this is more pronounced for *E. coli* (blue curve) than for *L. innocua* (red curve) (Fig. 5a,d). The energy cost for an *E. coli* and *L. innocua* cell to move from the ‘all in water’ phase to ‘all in air’ phase is 357 mJ and 136 mJ, respectively. A closer look at the energy curves where the bacterial cells are almost fully submerged in water (dashed squares in Fig. 5a), reveals an energy well for *L. innocua* at *z*_0_ = −0.9R, corresponding to an energy barrier (∆*G*_W_) of 0.93 mJ that a cell needs to overcome in order to fully immerse itself into the bulk water phase (Fig. 5b). Thus, at equilibrium, most of an *L. innocua* cell is immersed in water, but the cell remains interface-bound (Fig. 5d, red box). However, no energy well was found for *E. coli* cells anywhere on the curve (Fig. 5c).

### Effect of environmental conditions on post-UV cell repair

**Figure 6:**
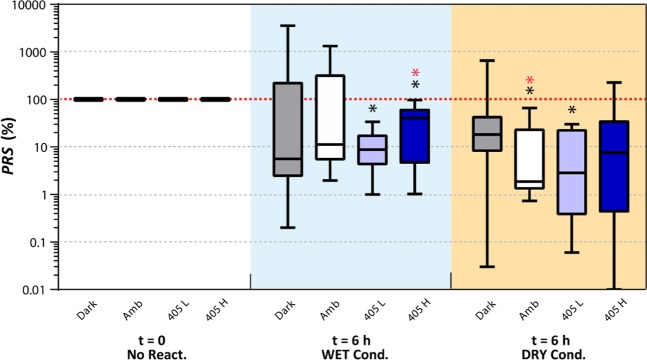
Box-and-whisker plot for post-reactivation survival (*PRS*) rate of *L. innocua* in the DRY and the WET condition under various lighting conditions: dark, ambient light (‘Amb’), low-intensity 405 nm LED (‘405 L’), and high-intensity 405 nm LED (‘405 H’). The line dividing each box into two parts represents the median, the top and the bottom of the boxes represent upper and lower quartiles, and the upper and the lower whiskers represent the max and the min values, respectively. Black asterisks denote significant difference in *PRS* compared to ‘100%’ without adjusting for multiple comparison (*p* < 0.05); red asterisks denote significance after adjusting for multiple comparison (*p*_adj_ < 0.05).

To evaluate the potential repair of bacterial cells under environmental conditions representative of real-world applications, *L. innocua* on a SS coupon was first exposed to the DUV LEDs, and subsequently subjected to various lighting and wetness conditions, and bacterial counts were determined after each treatment. Due to the large variability of the reactivation results, medians and interquartile range (depicted using a box-and-whisker plot) instead of means and standard deviations were used to represent the data, in order to avoid overall results being skewed by a few extreme values (Fig. 6). The median post-reactivation survival ratio (PRS) of *L. innocua* population was below 100%, regardless of the lighting and wetness conditions under which the reactivation process took place. It should be noted, however, that *PRS* > 100% did occur only rarely under certain reactivation conditions; more specifically: 3 out of the 11 biological replicates (i.e. 3/11) under the ‘Dark’-‘WET’ condition, 3/11 under ‘Amb’-‘WET’, 2/11 under ‘Dark’-‘DRY’, and 1/8 under ‘405 H’-‘DRY’. The ‘405 L’ was the only lighting condition that did not result in *L. innocua* reactivation during the 6 h period in either the ‘WET’ (*PRS* range: 0.06–30%) or ‘DRY’ (1–34%) conditions. This result is further substantiated by statistical analysis, which showed significantly lower *PRS* after reactivation under ‘405 L’ than before (*p* < 0.05, Wilcoxon signed-rank test). The greatest variability in reactivation was observed under the ‘Dark’-‘Wet’ conditions (*PRS* between 0.2–3556%). Because of the huge variability in data, no statistically significant differences among the medians from these eight reactivation conditions were found (*p* = 0.25, Kruskal-Wallis rank sum test).

## Discussion

DUV exposure is known to reduce microbial load on many fresh food products, including fruits, vegetables, juices, water, other processed food products, and processing equipment surfaces^18,19^. In this study, 5 log reduction of *E. coli* or *L. innocua* was achieved under a variety of surface conditions by exposure to DUV fluence between 1 to 10 mJ/cm^2^ (Figs. 1–3). These values are comparable to inactivation levels reported previously for aqueous suspensions of bacteria of 6–7 mm thickness, under constant stirring^9,10^. However, the fluence to achieve this level of inactivation was about 2 orders of magnitude higher than the fluence used to achieve similar inactivation values on agar surface by Kim *et al*.^14^. This difference could be caused by both the higher accessibility to DUV of the bacteria on the smooth agar surface, and the lower bacterial cell concentration used by Kim *et al*. compared to the current study.

When treating TLFs under static conditions, much higher inactivation efficiency was observed for the thinner TLFs than the thicker TLFs, at all fluence levels and for both microorganisms tested (Fig. 2b), which was attributed to the significant attenuation of DUV irradiance by the thicker TLFs (Fig. 2d). Yet, despite the limited penetration depth of DUV, up to 5 log reduction was achieved in the TLFs with thickness ≥1.2 mm (Fig. 2b). This suggests that there may be a constant movement of bacterial cells, either by passive diffusion or by active flagella propelled movement, which allows live cells to travel from the deep, low-exposure DUV zone into the ‘deadly’ high-exposure zone. This can explain the high level of bacterial death within the thick, highly light-absorbing bacteria suspension.

It has been previously reported that photoreactivation of DUV treated cells can occur after exposure to light in 300–500 nm wavelength range, due to repair of the UV damaged DNA^20^. In this study, there was no evidence of reactivation for cells exposed to 405 nm light post DUV exposure, under either DRY and WET conditions (Fig. 6). It was previously reported that exposure to 280 nm UV LEDs significant repressed photoreactivation of *E. coli*^9,11^. In the present study, although both dark and light repair were observed for *L. innocua* exposed to 280 nm DUV LEDs, the treatment caused irreversible damage to the bacterial cells. The 280 nm wavelength represents the peak absorbance for aromatic amino acids such as Tryptophan and Tyrosine^21^. This allows 280 nm DUV light to be absorbed by proteins, which eventually induces a higher level of deterioration of membrane proteins compared to shorter DUV wavelengths (i.e. the traditional germicidal 254 nm)^11,14^. In addition to DNA damage, 280 nm light can inflict ROS-mediated damage on multiple key bacterial targets, including membrane lipid peroxidation and respiratory enzyme activity^22^, physical membrane destruction^14^, and loss of membrane potential^14^. All these lead to broad-target disruption and lower the likelihood of full restoration of all key cellular functions by light or dark reactivation reactions.

Another important finding of this work is that *E. coli* and *L. innocua* cells spontaneously form a shell-like structure at liquid-air interfaces that form when a droplet of bacterial suspension is deposited on a solid substrate (Fig. 4b), whereas after the removal of the suspending liquid the bacterial population is distributed homogenously onto the solid substrate (Fig. 4d, lower panels). These two distinct types of organization of bacterial community, along with the low penetration depth of DUV in bacterial suspensions, as indicated by the sharp decline of ***E***_*λ=*280nm_ in Fig. 2d, inevitably result in the very different DUV irradiance distribution within a liquid droplet *vs* a liquid-less bacterial pellet. These in turn lead to substantial differences in inactivation kinetics in the two states for both *E. coli* (Fig. 3a) and *L. innocua* (Fig. 3b).

Both thermodynamic and bacterial mobility factors (e.g. flagella) may be responsible for the observed aggregation of bacterial cells at the liquid-air interface. From a thermodynamic viewpoint, the positioning of the first layer of *L. innocua* cells at the water-air interface can be explained by the energy well created across interfaces (Fig. 5b, right panel). The phenomenon of solid particles aggregation at the water - air interface is well known for hydrophobic abiotic microspheres in colloidal systems – phenomenon known as ‘Pickering Stabilization’^23,24^. Hence, it is not surprising that bacterial cells, which have a size at the upper end of colloidal particles, also tend to aggregate at liquid-air and liquid-liquid interfaces^25,26^, in an attempt to reduce the total energy of the system. Figure 5b indicates that the energy required to overcome the energy barrier facing the water phase is many orders of magnitude greater than *k*_B_*T*. Thus, it is extremely unlikely for cells to completely escape the water phase by just Brownian motion. Interestingly however, the model suggests that there is no energy well for *E. coli* cells near the water-air interface, and that it should be more energetically favorable for *E. coli* to remain in the water phase. *E. coli* cells are more hydrophilic than *L. innocua*^27,28^, and thus should have a lower tendency to aggregate at the interface than *L. innocua*^29^. Yet, the CLSM images obtained in this study show definite aggregation of the *E. coli* cells at the interface (Fig. 5b, left panel), despite the lack of an energy well at the interface. This discrepancy between the thermodynamic model prediction and the experimental result can be attributed to flagella-mediated clustering. Both Hsu *et al*.^28^ and Peel *et al*.^30^ showed that flagella are expressed by *E. coli* cells but not *L. innocua* grown at 37 °C. Chen *et al*.^31^ reported that actuated flagella can hydrodynamically induce bacterial aggregation of *Serratia marcescens*: an inward, tangential fluid flow within a liquid-air interface, created by flagella-propelled cells swimming toward the interface, can draw the neighboring cells in the interfacial plane together to form dynamic clusters. This clustering effect can be further strengthened by the formation of transient intercellular flagella bundles among adjacent cells^32^. Based on these prior studies it is reasonable to hypothesize that *E. coli* cells may form flagella-mediated layers at the liquid-air interface, despite the absence of an interface-bound energy well. Overall, our results imply that the formation bacterial layers at the water-air interface can be driven by thermodynamics and/or bacterial surface appendages, and the relative contribution of these two factors could vary greatly depending on the cells’ physicochemical properties (e.g. hydrophobicity) and physiology (e.g. expression of surface appendages). Future work is needed to investigate aggregation phenomena at various liquid-air interfaces of a wide range of microbial species and strains that have been implicated in foodborne outbreaks and nosocomial infections.

The deviation of inactivation kinetics on SS from the Weibull model under wet conditions, but not the dry conditions, for which an extremely good fit of the model was obtained (Fig. 3c,d), is rather intriguing. The Weibull model is well-suited for describing the inactivation kinetics of one bacterial population, with normally distributed susceptibility to a given stimulus (e.g. DUV)^33^. In case of the wet SS coupons, the bacterial layers at the liquid-air interface function as a protective shell, effectively reducing the intensity of DUV that is reaching the planktonic bacteria inside the liquid droplet. The co-existence of a population of packed cells exposed to high DUV radiance, and a population of planktonic cells exposed to a much lower level of DUV is likely the cause of the deviation of the experimental results on wet SS coupons from the predictions by the Weibull model (Fig. 3e,f). It should be noted that the agreement between the Weibull predictions and the results in TLFs is also very good (Table 1).

This study and previous studies prove the true potential of DUV LEDs for bacterial inactivation, both in liquid and on solid substrates, and an exposure time of about 10 min is sufficient to achieve a 5-log reduction for the bacteria and disinfection scenarios tested here. Microbial contamination and growth are most problematic in certain hotspots where moisture and nutrients are readily accessible, and hence targeting DUV treatment at these high-risk surfaces will be of highest practical advantage. These findings are based on work performed with a single bacterial strains, so that the effects of substrate and environmental factors can be separated from biological factors. To further validate these findings, future studies on microbial decontamination by DUV LEDs may consider testing a broader range of challenge organisms and/or strain cocktails, so thatto also account for inter- and intra-species variability^34^.

## Conclusions

In this work, a proof-of-concept panel comprising an array of LEDs emitting DUV of 280 nm wavelength was assembled, and its inactivation capability tested against surrogates for pathogenic *E. coli* and *L. innocua*, on substrate types frequently encountered in environmental, clinical, and food processing and handling environments. The tested DUV LEDs demonstrated very promising surface disinfection efficiency in all the scenarios tested, with no detectable survivors for either strain after 5 min (or 11.9 mJ/cm^2^) treatment on nutrient-rich surfaces, and 5-log reduction within 10 min (or 23.8 mJ/cm^2^) on SS under both wet and dry conditions, and in TLFs of thickness less than 0.6 mm. DUV inactivation of bacteria suspensions in liquid films thicker than 1.2 mm was much slower, due to the low penetration depth of DUV through concentrated bacterial suspensions. Furthermore, it was proven here that aggregation of bacterial cells at the liquid-air interface, driven by thermodynamic and motility factors, shielded the cells inside of the liquid droplets from the bactericidal DUV, which slowed down the initial stages of inactivation. Post DUV treatment exposure to low-intensity 405 nm lighting provided consistent inhibition of reactivation.

This work also provides insights into how to select DUV LED treatment parameters in order to achieve effective and efficient surface disinfection, with broad implications on the disinfection of both biotic and abiotic surfaces. With the advent of robotics and computer vision technologies, it is now possible to design LEDs-integrated robotics to deliver the appropriate level of DUV energy to contaminated, high-risk, surfaces, in a targeted manner, which can become more energy efficient and sustainable than traditional DUV technology.

## Materials and Methods

### Bacterial cultures

#### Listeria innocua

FSL C2-008 (environmental isolate from a smoked fish plant) and *Escherichia coli* ATCC 25922 (American Type Culture Collection, Manassas, VA) were stored in glycerol stock solution at −80 °C prior to use. Culture reactivation was conducted by first streaking the frozen culture on Trypticase Soy Agar (TSA; BD Difco, Franklin Lakes, NJ) and incubate (37 °C, 24 h) to obtain isolated colonies, followed by loop-inoculation in 3 mL Tryptic Soy Broth (TSB; BD Difco, Franklin Lakes, NJ) for passage one (37 °C, 24 h), and passing 30 µL of grown passage one culture to fresh 3 mL TSB for passage two (37 °C, 16 h). To replace TSB with UV-transmitting phosphate buffer (Supplementary Fig. S1), the resulting early stationary phase culture were centrifuged (5000 RPM, 10 min, 21 °C) and resuspended in sterile Butterfield Phosphate Buffer (BPB, pH = 7.2). This wash step was repeated two more times to ensure minimal remnants of TSB in the final bacteria suspension. The final concentration of bacteria suspended in the BPB was about 10^9^ CFU/mL for both strains.

### DUV LED treatment

**Figure 7:**
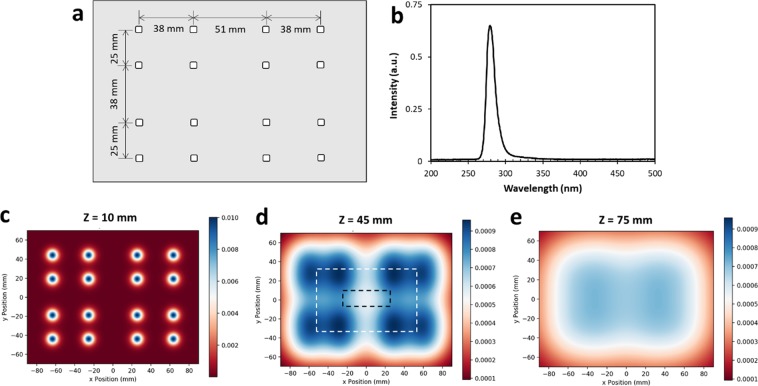
The custom-made DUV LED panel and its performance characteristics. (**a**) Arrangement of DUV LEDs on the panel. (**b**) Typical emission spectrum of the DUV LEDs. (**c–e**) Simulation of the distribution of DUV energy received by a flat surface located at 10 mm, 45 mm, 75 mm away from the light source, respectively. A larger intensity scale was used for (**c**) than (**d**) and (**e**), to capture the details of the distribution. The dashed-line rectangles illustrate the position of a liquid chamber or a SS coupon, respectively, in the DUV energy field.

A custom-made DUV LED chamber was used to perform all inactivation experiments. The apparatus delivers DUV light via 16 individual DUV LEDs (SMD3535, TaoYuan Electron Ltd., Shenzhen, China) arranged as shown in Fig. 7a. These LEDs produce a monochromatic emission spectrum with the peak at 280 nm (Fig. 7b). To determine an appropriate separation distance between the DUV LED panel and target surfaces, spatial distribution of 280 nm irradiation maps at the target surfaces were simulated for separation distances of 10 mm (Fig. 7c), 45 mm (Fig. 7d), and 75 mm (Fig. 7e). A separation distance of 45 mm was selected for all DUV exposure experiments because it provided a good balance between the intensity of the irradiance and the homogeneity of the distribution. At this distance, when the power source was operated under CV mode, with the potential set to 5.30 V, resulting in a current of ~320 mA, the fluence rate below the middle of the panel was 40 µW/cm^2^.The entire apparatus was covered with aluminum foil to isolate the DUV treatment from any potential disturbance by ambient light. Meanwhile, as a safety precaution, the aluminum foil blocked the UV light from human operators during the treatment. Additionally, on rare occasions when direct observation of the operating DUV LEDs was necessary, the operators were required to ware UV protection safety goggles. The following treatment durations were chosen to deliver different UV doses: 60 s, 180 s, 300 s, 600 s, 1000 s, corresponding to cumulative UV fluence of 2.38 mJ/cm^2^, 7.13 mJ/cm^2^, 11.88 mJ/cm^2^, 23.76 mJ/cm^2^, and 39.60 mJ/cm^2^.

### Bacteria Inactivation by DUV LED on various substrates

#### Nutritive agar plate treatments

Both the *E. coli* and *L. innocua* cultures were streaked in two parallel lines on TSA plates of 100 mm in diameter. Half of each plate was covered with aluminum foil, to divide the surface of the plate and the bacterial streaks into a ‘Exposed’ section and a ‘Covered’ section (Fig. 1a), followed by exposure to DUV for a specified duration (10 s to 25 min). After DUV treatment, the plates were incubated at 37 °C for 24 h for qualitative evaluation of bacteria inactivation.

#### Thin liquid films (TLF) treatments

Liquid chambers (Lab-Tek II Chamber Slide chamber, 17 mm × 48 mm; Fisher Scientific, Rochester, NY) were used to hold *E. coli* or *L. innocua* suspensions in the form of TLFs to mimic contaminated standing water. Before the experiment, the liquid chambers were decontaminated by soaking in 70% ethyl alcohol (Fisher Scientific, Rochester, NY) for 24 h, followed by drying in a biosafety cabinet for 2 h to evaporate the remaining ethyl alcohol. After that, bacteria suspensions were pipetted into the liquid chambers and allowed to equilibrate for 3 min before starting the DUV exposure. The effect of strains on DUV inactivation kinetics was investigated by exposing 1 mL of liquid film (thickness = 1.2 mm) containing either *L. innocua* or *E. coli*, for the durations specified previously. To determine the effect of thin film thickness on inactivation kinetics, 0.5 mL, 1 mL, 1.5 mL, and 2 mL of *L*. *innocua* suspension was aliquoted into a liquid chamber, resulting in liquid film thickness of 0.6 mm, 1.2 mm, 1.8 mm, and 2.4 mm, respectively, followed by 3-min equilibration and subsequent DUV LED treatment for various durations. Due to the concave meniscus exhibited by the liquid films at the liquid chamber walls, the average thickness of the bacteria containing liquid films was calculated by dividing the volume of bacteria suspension by the bottom area of the liquid chamber instead of being measured directly. The bacteria suspension from both UV-treated and non-treated control samples were serially diluted with BPB and enumerated using standard plate counting method on TSA agar. Total bacteria reduction was calculated using the following equation:

1 $$Log\,Reduction={\mathrm{Log}}_{10}(\frac{N}{{N}_{0}})$$

where *N*_0_ and *N* are the bacteria counts (in colony forming units per mL of suspension, CFU/mL) before and after DUV treatment, respectively. The detection limit of the inactivation experiments in TLF is 10 cells per mL of bacteria suspension, for both *E. coli* and *L. innocua*.

All DUV LED treatments were performed in triplicate, with independently grown bacterial cultures used in each replicate.

Transmittance spectra of DUV through TLFs of thickness of 0.6 mm, 1.2 mm, 1.8 mm, and 2.4 mm were determined using an HR2000CG-UV-NIR spectrometer equipped with a DH-2000-BAL UV-VIS-NIR light source (Ocean Optics Inc, Largo, FL, USA). Briefly, *E. coli* and *L. innocua* suspensions (prepared as described above) were aliquoted into a space of desired thickness, as specified above, which was created by inserting polyethylene spacers between two clean DUV transmitting fused quartz slides (Grade GE124, Technical Glass Products, WA, USA), placed horizontally on a metal stand. The optical fiber outlet (connected to light emitter) and the receiver inlet (connected to the detector) were perpendicularly affixed to the top and bottom quartz slide, respectively. An illustration of the setup is shown in Supplementary Fig. S3. The volume of the suspensions was adjusted to ensure that it covered the entire cross-section of the beam emitted by the fiber outlet (about 4 cm^2^ surface area). All transmittance measurements were taken immediately after the suspensions were aliquoted, to minimize the potential interference by bacterial attachment onto the slides. Technical duplicates were performed on each sample to account for potential variation caused by the positioning of the suspension with respect to the beam.

#### Stainless-steel coupon treatments

Food-grade SS coupons (50 mm × 100 mm) with a glass bead blasted finish (*R*_a_ = 0.78 µm) were used to simulate polished SS surfaces commonly encountered in food handling and medical environments^35^. To remove any surface chemical contaminants, the SS coupons were sequentially submerged in a rotating bath (100 RPM) of 95% acetone (Fisher Scientific, Rochester, NY), 95% ethyl alcohol and deionized water, for 10 min at each step. The coupons were then autoclaved (15 min, 121 °C) in individually sealed sterilization pouches to kill any potential microbial contaminants. The SS coupons prepared as described here exhibited a mean water contact angle of 62.2 ± 3.3°, as determined by a static sessile drop method with a Rame-Hart 500 goniometer (Rame-Hart Inc., Succasunna, NJ, USA).

For inoculation, a total of 1 mL bacteria suspension was deposited on the SS surfaces as 20 evenly spaced droplets of 50 µL each. The inoculated coupons were then divided into two treatment groups, as follows: 1) for the wet condition, inoculated coupons were left to equilibrate in the laminar flow hood (23 °C, relative humidity = 17%) for 3 min; 2) for the dry condition, the inoculated coupons were left to dry under the hood for about 3 h, until they reached a constant weight, without excessive drying (Supplementary Fig. S4).

The SS coupons prepared as described above were subjected to DUV LED treatments, as described in section 2.3.1, after which they were individually placed in sterile Whirl-Pak bags with 100 mL BPB and sonicated for 5 min at 40 kHz (Branson 1210 Ultrasonic Cleaner, Branson Ultrasonics, Danbury, CT). This method of recovery was used since Bjerkan *et al*.^36^ showed that ultrasonication (>20 kHz) for 5 min achieved the highest recovery of bacteria from metal plates compared to other commonly used methods such as manual scraping. Preliminary experiments conducted as part of the present study also showed no effect of ultrasonication for 5 min on bacterial viability (data not shown). After sonication, the BPB that contained recovered cells was subjected to serial ten-fold dilutions with sterile BPB, spread plating on TSA plates, and enumeration of CFUs after incubation at 37 °C for 24 h. The control followed the same recovery and enumeration procedures except for the DUV LED treatment. The detection limit of the inactivation experiments on SS surfaces was 100 cells per SS coupon (50 cm^2^) for both *E*. *coli* and *L*. *innocua*. Inactivation effectiveness, expressed in Log reduction, was determined using Eq. 1. All DUV LED treatments were performed in triplicate, with independently grown bacterial cultures.

### Reactivation of DUV treated bacteria

To evaluate potential reactivation of DUV treated bacteria under conditions mimicking real-world applications, *L. innocua* was spot-inoculated onto the SS coupons, exposed to DUV under the wet condition for 600 s (or 23.76 mJ/cm^2^), and then subjected to a 6-h reactivation step under four different lighting conditions: (1) Dark; (2) white ambient light (60 W, GreybaR Electrics, Philadelphia, PA), labeled ‘Amb’; (3) low luminance flux (260–290 µW/cm^2^) and (4) high luminance flux (490–610 µW/cm^2^) 405 nm LEDs (Vital Vio, Troy, NY), labeled ‘405 L’ and ‘405 H’, respectively. A 6-h reactivation period was used because previous studies showed that the post-UV bacterial reactivation plateaus around this time^9^. Reactivation under each lighting condition was also tested under the ‘DRY’ or the ‘WET’ condition (‘DRY’ and ‘WET’ were capitalized to be differentiated from the dry and the wet conditions used in the kinetics study). For reactivation under ‘DRY’ conditions, the DUV treated bacterial inoculum was allowed to dry on the SS coupons in a laminar flow hood for 6 h, followed by the same recovery and enumeration steps described in 2.3.

For the ‘WET’ condition, the UV-treated bacteria on the SS coupons were first transferred into BPB following the same procedures as for recovery. To conduct the ‘Dark’ reactivation under the ‘WET’ condition, 1 mL of the resulting suspension was added to opaque 1.5 mL Eppendorf tubes (Eppendorf Flex-Tubes, Hauppauge, NY). To conduct the ‘Amb’, ‘405 L, and ‘405 H’ reactivation, 1 mL of the suspension was added to a custom-made chamber covered with an UV-transmitting quartz window lid, and all sides sealed with Parafilm to prevent evaporation of the liquid during reactivation. All ‘WET’ samples were kept at 21 °C for 6 h under the specified lighting conditions before enumeration.

For each of the 8 reactivation conditions tested (2 wetness conditions × 4 lighting conditions), the bacterial concentration at the end of the UV treatment (*N*_UV_) and post-reactivation (*N*_PR_) were determined by standard plate counting on TSA plates after incubation for 24 h at 37 °C. The percent post-reactivation survival ratio (*PRS*) for each individual biological replicate was calculated using the equation:

2 $$PRS=(\frac{{N}_{PR}}{{N}_{UV}})\cdot 100 \% $$

*PRS* greater than 100% represents an increase in survivor counts due to reactivation of injured cells, while PRS equal to or less than 100% represents no change, or a further decrease in counts during the resuscitation process. To account for potential genetic diversity within the strain, a larger number of biological replicates (6 ≤ n ≤ 11) were included than in the previous experiments, in order to acquire representative, unbiased reactivation results.

### Modelling of inactivation kinetics

Microbial inactivation by DUV LED was modelled by the Weibull model^37^:

3 $${\log }_{10}(N/{N}_{0})=\alpha {t}^{\beta }$$

where $N/{N}_{0}$ represents the ratio of survivors after treatment (*N*) over the initial population (*N*_0_), *α* is the scale parameter and *β* is the shape factor. The non-linear regression was performed using the statistical software R (R Foundation for Statistical Computing, version 1.1.463).

### Confocal laser scanning microscopy (CLSM) imaging

The distribution of *E. coli* and *L. innocua* cells within liquid droplets and dried pellets on the SS coupons was visualized using a Zeiss LSM 710 equipped with inverted immersion objectives (Carl Zeiss, Jena, Germany), as described elsewhere^27,38^. Briefly, to image a bacteria-containing liquid droplet, 5 µL bacteria-BPB suspension (prepared as described above), stained with a LIVE/DEAD *Bac*Light fluorescent dye (Thermo Fisher Scientific, Waltham, MA, USA), was aliquoted onto the glass bottom of a confocal-compatible dish (Mat-Tek Corporation, Ashland, MA, USA). The scanning process was performed *in situ*, with minimal mechanical disturbance on bacterial movement and distribution. Apochromat 10 × and 40 × water immersion objective lenses were used for capturing the overall distribution and the details at the liquid-air interfaces, respectively. The Z-scan mode was deployed to capture the cell distributions in the three-dimensional space.

To image a dried bacteria pellet, 50 µL of bacterial suspension was aliquoted onto a smaller version (1 in × 2 in) of the SS coupon used in the inactivation experiments, followed by drying for 3 h. After drying, diluted fluorescent dye (5 µL in 3 mL BPB) was pipetted onto the dry bacteria pellet, incubated for 20 min in the dark at 21 °C, and the unbound dye was rinsed off gently with a 0.15 M NaCl solution. Next, the SS coupon was inverted and placed on the glass bottom of the dish, with the pellet side facing downward and immersed in *Bac*Light mounting oil. Z-scans were performed throughout the entire thickness of the pellet.

Considering that sample preparation procedures for confocal microscopy could result in biased conclusions on bacterial viability, only the signal from the green fluorescent channel, which accounts for all cells, regardless of their viability, was shown and discussed in the paper.

### Thermodynamic modelling of bacterial cell distribution at the liquid-air interfaces

A thermodynamic model was developed to predict the distribution of bacteria at the liquid-air interface. For accurate representation of the morphology of the *E. coli* and *L. innocua* cells, bacterial cells were modeled as hemisphere-capped cylindrical rods. Neither bacterial appendages, nor active cellular motions were included in this model, and their roles will be discussed separately. Only the bacteria that oriented parallel to the interface were considered because it was previously shown that this orientation is thermodynamically favored and therefore most likely to be adopted by the cells at equilibrium^39^.

The total surface energy of the system, *G*, which comprises the surface energy contributed by the rod-shaped bacterial cells (subscript ‘b’), air (subscript ‘a’), and water (subscript ‘w’) – was derived as a function of a dimensionless coordinate, *z*_0_:

4 $$G({z}_{0})={G}_{cap}({z}_{0})+{G}_{cyl}({z}_{0})$$

4a $${G}_{cap}({z}_{0})=2\pi {R}^{2}{\gamma }_{bw}(1+{z}_{0})+2\pi {R}^{2}{\gamma }_{ba}(1-{z}_{0})-\pi {R}^{2}{\gamma }_{aw}(1-{z}_{0}^{2})$$

4b $${G}_{cyl}({z}_{0})=[2\pi -2co{s}^{-1}(\,-\,{z}_{0})]R(l-2R){{\rm{\gamma }}}_{bw}+2co{s}^{-1}(\,-\,{z}_{0})R(l-2R)\,{{\rm{\gamma }}}_{ba}-2R\sqrt{1-{{z}_{0}}^{2}}(l-2R){{\rm{\gamma }}}_{wa}$$

where ${G}_{cap}$ and ${G}_{cyl}$ are the surface energy contributions from the hemispherical caps and the cylinder connecting the two caps; ${\gamma }_{bw}$, ${\gamma }_{ba}$, and ${\gamma }_{aw}$ are interfacial energy between bacteria and water, bacteria and air, and water and air, respectively; ${z}_{0}$ is the vertical coordinate of the center of the bacterial cell in relation to the water level, $\,z$, normalized by the radius of the cell, $R$; $l$ is the length of the cell. The values of ${\gamma }_{bw}$, ${\gamma }_{ba}$, and ${\gamma }_{aw}$ were calculated from the contact angles of three probe liquids on bacterial cell lawns, as described elsewhere^27,40^. The contact angle values and bacterial cellular dimensions used in the model are summarized in Supplementary Table S1.

### Statistical analysis

Analysis of variance and post hoc Tukey’s HSD were used to compare experimental and Weibull calculated inactivation data. Non-parametric statistics were used to analyze the reactivation results because *PRS* data did not follow a Gaussian distribution. Specifically, the Kruskal-Wallis rank sum test was used to compare the *PRS* from the eight reactivation conditions and the Wilcoxon signed-rank test (two-sided) was used to evaluate the significance of the change in *PRS* before and after reactivation under each of the eight conditions. The False Discovery Rate method was used to determine the adjusted *p*-value for multiple comparisons (n = 8). A confidence level of 95% is adopted for all statistical tests. All statistical tests were performed using the statistical software R (version 1.1.463).

## Supplementary information

Supplementary information.

## Data Availability

The data sets generated and analyzed during the current study are available from the corresponding author on reasonable request.
