# Supplementary material for: Inactivation of Listeria and E. coli by Deep-UV LED: effect of substrate conditions on inactivation kinetics
Source: Sci Rep. 2020 Feb 25;10:3411. doi: 10.1038/s41598-020-60459-8 (PMC7042228; doi:10.1038/s41598-020-60459-8)
Supplement: Supplementary file 1 — Supplementary information. [file 41598_2020_60459_MOESM1_ESM.pdf]

Supplementary Information

Inactivation of *Listeria* and *E. coli* by Deep-UV LED: effect of substrate conditions on inactivation kinetics

Authors: Yifan Cheng[1†], Hanyu Chen[1†], Luis Alberto Sánchez Basurto[2], Vladimir V. Protasenko[3], Shyam Bharadwaj[3], Moududul Islam[3], Carmen I. Moraru[1*]

[1]Department of Food Science, Cornell University, Ithaca, NY 14850, U.S.A

[2]Universidad Autónoma de Querétaro, Santiago de Querétaro, México

[3]Electrical and Computer Engineering, Cornell University, Ithaca, NY 14850, U.S.A

[*] Corresponding author. Email: cim24@cornell.edu

[†] These authors contribute equally to this work

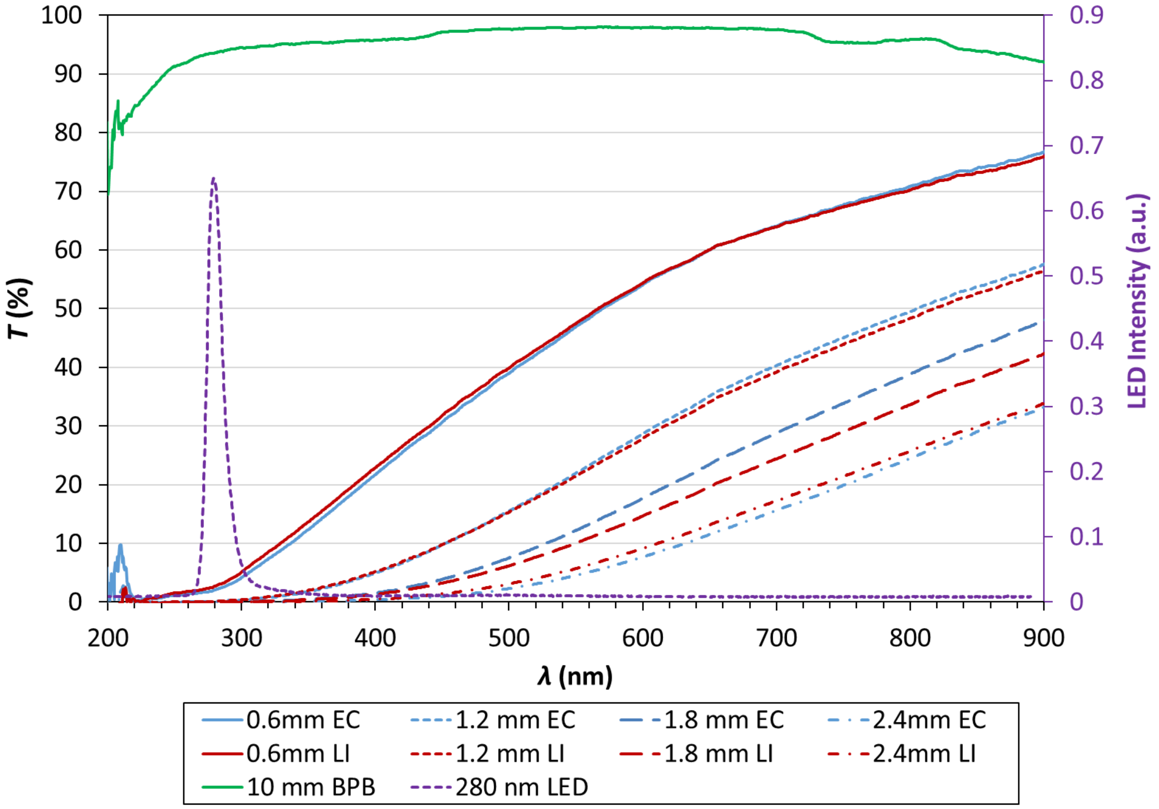

Figure S1. Full transmittance spectra of *E. coli* (EC, blue) and *L. innocua* (LI, red) suspensions of various thickness, BPB medium, and the emission spectrum of the DUV LED used in this work.

Table S1. Summary of the bacterial cellular dimensions and contact angle values used in the thermodynamic model

Bacterial features	Mean ± SD for indicated bacteria strains		Reference
	E. coli ATCC 25922	*L. innocua* FSL C2-008	
Bacterial dimensions (µm) [a]			
Length - *l*	2.22 ± 0.29	1.26 ± 0.15	[1]
Radius - *R*	0.32 ± 0.02	0.26 ± 0.02	
Contact angles (°) [b]			
Water	40.3 ± 1.1	46.5 ± 1.2	[2]
Glycerol	70.7 ± 2.1	65.9 ± 2.3	
Diiodomethane	56.9 ± 3.0	46.4 ± 1.7	

[a] Bacterial dimensions were measured on calibrated SEM images of cells on silica substrates

[b] Contact angles were measured on bacterial cell lawns collected on filter paper

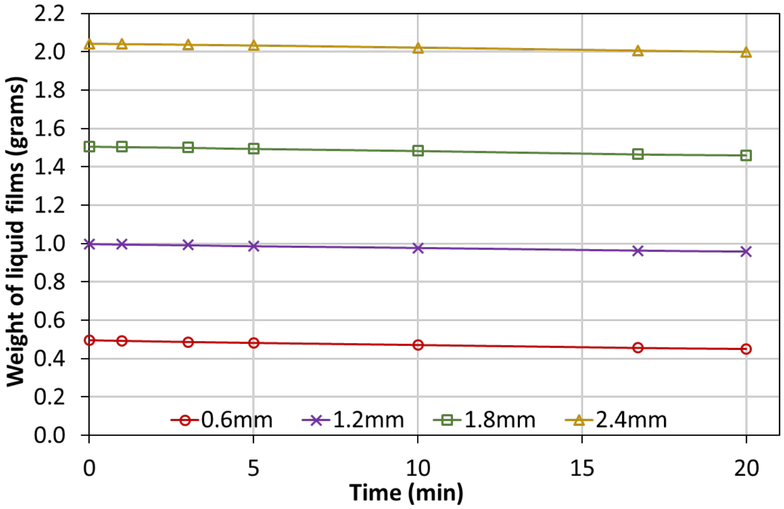

Figure S2. Evaporation of thin liquid films of various thickness monitored as weight loss over time.

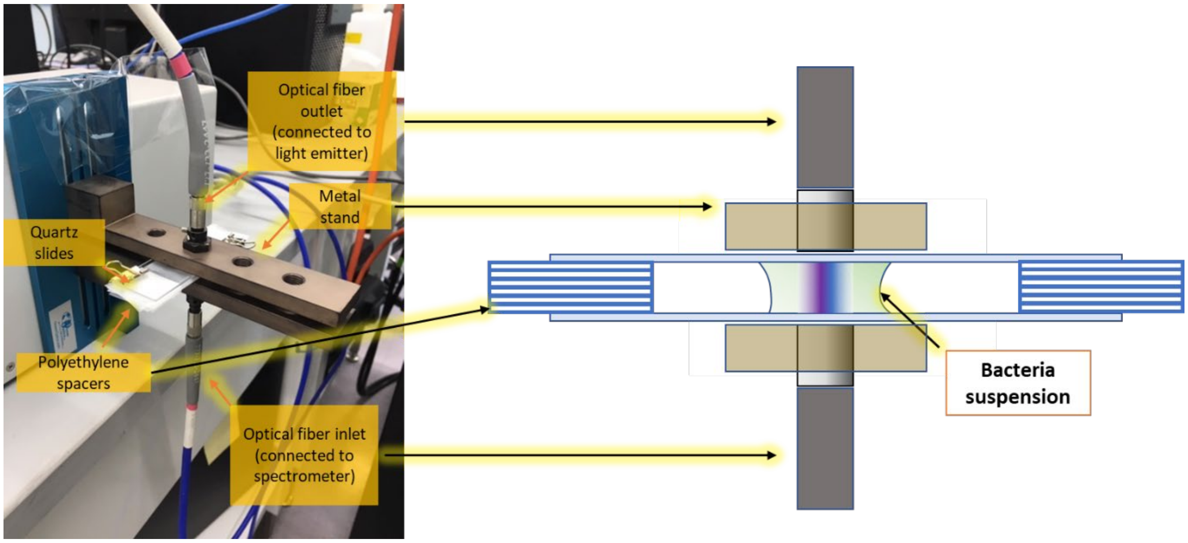

Figure S3. Photographic and schematics illustration of the experimental setup for measuring DUV transmittance. Thickness of the bacteria suspension was adjusted by changing the number of polyethylene spacers flanked by the quartz slides.

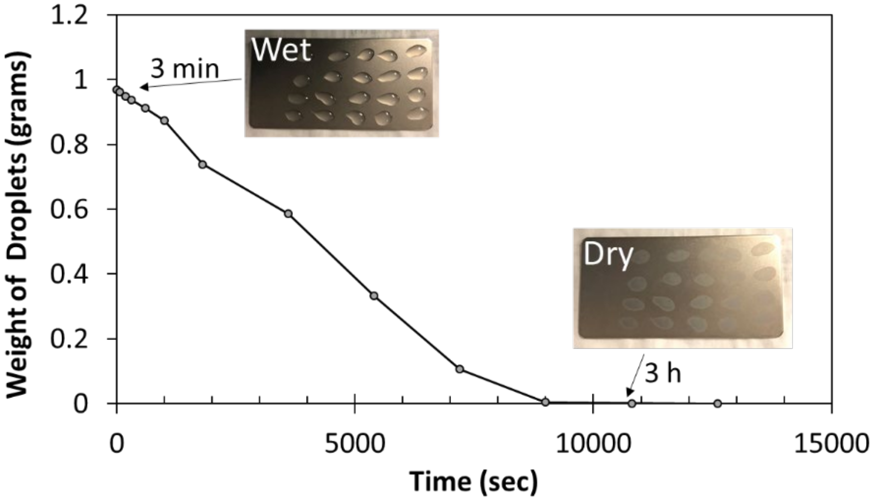

Figure S4. Kinetics of drying of the inoculated SS coupons and selection of the dry and the wet conditions used for the DUV LED inactivation experiments.

References

1. Hsu, L., Fang, J., Borca-Tasciuc, D., Worobo, R. & Moraru, C. I. The effect of micro- and nanoscale topography on the adhesion of bacterial cells to solid surfaces. Appl. Environ. Microbiol. (2013). doi:10.1128/AEM.03436-12

2. Feng, G. et al. Alumina surfaces with nanoscale topography reduce attachment and biofilm formation by *Escherichia coli* and *Listeria* spp. Biofouling 30, 1253–1268 (2014).
